# Transcatheter aortic valve replacement in atypical valve anatomy using the Lotus valve: A Chinese single-center experience

**DOI:** 10.1007/s00059-018-4778-z

**Published:** 2019-01-29

**Authors:** Q. Xu, X. Liu, J. Jiang, Y. He, Q. Zhu, F. Gao, F. Du, W. He, J. Cheng, M. Kong, Z. Pu, Q. Zhou, R. Gooley, J. Wang

**Affiliations:** 1 Department of Cardiology, The Second Affiliated Hospital, Zhejiang University School of Medicine, 310009 Hangzhou, China; 2 Department of Anesthesia, The Second Affiliated Hospital, Zhejiang University School of Medicine, 310009 Hangzhou, China; 3 Department of Cardiac Surgery, The Second Affiliated Hospital, Zhejiang University School of Medicine, 310009 Hangzhou, China; 4 Department of Radiology, The Second Affiliated Hospital, Zhejiang University School of Medicine, 310009 Hangzhou, China; 5 Monash Cardiovascular Research Centre, Monash University, Victoria, Clayton Australia

**Keywords:** Aortic valve stenosis, Transcatheter aortic valve implantation, Lotus aortic valve, Bicuspid aortic valve, Heart valve diseases, Aortenklappenstenose, Transkatheter-Aortenklappenimplantation, Lotus-Aortenklappe, Bikuspide Aortenklappe, Herzklappenerkrankungen

## Abstract

**Background:**

In the West, the safety and efficacy of the Lotus valve have been demonstrated; however, data in the Chinese population are still lacking. Few studies have compared the clinical outcomes of transcatheter aortic valve replacement (TAVR) with the Lotus valve in patients with bicuspid or tricuspid aortic valve stenosis. Our aim was to assess TAVR outcomes with the Lotus aortic valve in a Chinese patient cohort.

**Methods:**

In total, 23 symptomatic, high-surgical risk patients with severe aortic valve stenosis were enrolled. Among them, nine patients (39%) had bicuspid aortic valves, and three patients had a large annulus dimension. The Lotus valve was successfully implanted in all patients. To facilitate accurate positioning, partial re-sheathing was attempted in ten patients (43.5%), while one patient had a full retrieval. One-year clinical follow-up was completed in all patients.

**Results:**

There were no deaths, strokes, or major adverse cardiac and cerebrovascular events in 22 of the 23 patients at 30 days; the all-cause mortality rate at 1 year was 4.4% (1 of 23 patients). The mean aortic valve gradient decreased from 51.5 ± 8.8 mm Hg at baseline to 13.4 ± 4.9 mm Hg (*p* < 0.001) and the valve area increased from 0.6 ± 0.2 cm^2^ to 1.5 ± 0.4 cm^2^ (*p* < 0.001) at 30 days. Paravalvular leakage was absent or mild (22%), and no patient had severe paravalvular leakage. Six patients (26.1%) required a postprocedural pacemaker. There was no difference regarding the procedural and the 1‑year outcomes between patients with bicuspid and tricuspid aortic valve stenosis.

**Conclusion:**

Our single-center experience demonstrated that the Lotus valve is feasible and effective for Chinese patients with aortic valve stenosis, including atypical cases with bicuspid aortic valves or large aortic annulus size.

## Abstract (de)

**Hintergrund:**

In der westlichen Welt wurden Sicherheit und Wirksamkeit der Lotus-Klappe nachgewiesen, für die chinesische Bevölkerung fehlen solche Daten jedoch. Nur in wenigen Studien wurden die klinischen Ergebnisse des Transkatheter-Aortenklappenersatzes (TAVR) mit der Lotus-Klappe bei Patienten mit Stenose einer bikuspiden oder trikuspiden Aortenklappe verglichen. Ziel war es, die Ergebnisse der TAVR mit der Lotus-Aortenklappe in einer chinesischen Patientenkohorte zu untersuchen.

**Methoden:**

In die Studie wurden 23 Patienten mit symptomatischer schwerer Aortenklappenstenose und hohem Operationsrisiko einbezogen. Davon hatten 9 Patienten (39 %) eine bikuspide Aortenklappe, bei 3 Patienten lag ein großer Aortenklappenring vor. Die Lotus-Klappe wurde bei allen Patienten erfolgreich implantiert. Zur Erleichterung der genauen Platzierung wurde bei 10 Patienten (43,5 %) ein partielles Zurückführen der Klappe in den Einführkatheter erprobt, in einem Fall erfolgte die Wiederentfernung der Klappe. Bei allen wurde die Nachbeobachtung über ein Jahr komplett durchgeführt.

**Ergebnisse:**

Bei 22 der 23 Patienten gab es nach 30 Tagen weder einen Todesfall, noch kam es zum Schlaganfall oder schweren unerwünschten kardialen oder zerebrovaskulären Ereignissen; die Rate der Mortalität jeglicher Ursache nach einem Jahr betrug 4,4 % (1 von 23 Patienten). Der mittlere Aortenklappengradient nahm von 51,5 ± 8,8 mm Hg zu Beginn auf 13,4 ± 4,9 mm Hg (*p* < 0,001) ab, und die Klappenöffnungsfläche nahm von 0,6 ± 0,2 cm^2^ auf 1,5 ± 0,4 cm^2^ (*p* < 0,001) nach 30 Tagen zu. Paravalvuläre Lecks traten nicht oder nur leichtgradig auf (22 %), in keinem Fall kam es zum schweren paravalvulären Leck. In 6 Fällen (26,1 %) war nach dem Eingriff eine Schrittmacherimplantation erforderlich. Zwischen Fällen mit Stenose einer bikuspiden und einer trikuspiden Aortenklappe bestand kein Unterschied beim prozeduralen und Einjahresergebnis.

**Schlussfolgerung:**

Diese Erfahrungen des Einzelzentrums der Autoren zeigten, dass die Lotus-Klappe für chinesische Patienten mit Aortenklappenstenose, einschließlich atypischer Fälle mit bikuspider Aortenklappe oder großem Aortenringdurchmesser, geeignet und wirksam ist.

Transcatheter aortic valve replacement (TAVR) has become a standard treatment for severe symptomatic aortic stenosis (AS) in patients who are at intermediate-to-high risk for surgical valve replacement [1–5]. However, moderate or severe postprocedural paravalvular leakage (PVL) is considered a significant independent predictor of acute and long-term mortality [6–10].

The fully repositionable and retrievable transcatheter Lotus aortic valve has a unique adaptive seal designed to minimize PVL. The valve is deployed via controlled mechanical expansion, which allows for precise positioning. The safety and efficacy of the Lotus valve have been demonstrated in patients with severe AS at high or intermediate surgical risk in the Western world [11–15]. However, data about the use of the Lotus valve for Chinese patients are still lacking. Some studies reported that TAVR with the Lotus valve is feasible for bicuspid AS [16, 17]. Nevertheless, there are limited data comparing the Lotus valve in bicuspid and tricuspid AS.

Herein, we report on the 1‑year clinical outcomes with the Lotus valve in the prospective LOTUS-CHINA study. This study was designed to assess the safety and efficacy of the Lotus valve system for patients with severe AS in a Chinese patient cohort (ClinicalTrials.gov registration number NCT02536703).

## Methods

### Study design and patient population

The LOTUS-CHINA trial is a prospective, open-label, single-arm study. Between November 2015 and January 2017, 23 patients diagnosed with severe AS were enrolled in this study. All patients were classified as New York Heart Association (NYHA) functional class ≥ II, had symptoms attributable to severe AS such as angina pectoris or syncope and had transthoracic echocardiography (TTE) measurements consistent with severe AS (aortic valve area [AVA] < 1.0 cm^2^, and a mean aortic pressure gradient [PGmean] > 40 mm Hg or a peak systolic velocity [Vmax] > 4 m/s). Patients were considered to be at high risk on the basis of a Society of Thoracic Surgery (STS) score of ≥ 8% [18] and if a multidisciplinary heart team—including an interventional cardiologist, a cardiothoracic surgeon, and an anesthetist—agreed that the subject was likely to benefit from TAVR, was frail and/or had comorbidities associated with high surgical risk.

Data collection was followed by a study protocol that was approved by the local ethics committee, and registered with ClinicalTrials.gov (NCT02536703). All enrolled patients gave written informed consent.

Patient characteristics including age, gender, clinical symptoms, STS score, comorbidities etc. were collected. Baseline TTE measurements were collected including AS severity (AVA, PGmean, and Vmax), left ventricular function, and pulmonary pressure. Patient inclusion and exclusion criteria were followed as per the published protocol on ClinicalTrials.gov (NCT02536703). Procedural results as well as 30-day and 1‑year outcomes were measured according to the Valve Academic Research Consortium (VARC)-2 criteria [19].

The primary endpoints were all-cause mortality, myocardial infarction, and stroke at 30 days after the procedure. Procedural complications, functional status (NYHA classification), and echocardiographic prosthesis status at 30 days and 1 year were reported as the secondary endpoints. Additionally, the study also compared procedural characteristics and postprocedural outcomes between tricuspid aortic valves (TAVs) and bicuspid aortic valves (BAVs).

### Device characteristics

The Lotus valve system (Boston Scientific Corporation, Natick, MA, USA) consists of a bioprosthetic aortic valve (a braided nitinol wire frame with three bovine pericardial leaflets) premounted on a preshaped delivery catheter. The valve is deployed via controlled mechanical expansion, which enables predictable and precise placement. The lower half of the Lotus valve is surrounded by a polymer membrane designed to fill the space between the native annulus and the prosthetic valve frame, thereby reducing PVL. The Lotus valve begins functioning early in the deployment process, providing hemodynamic stability and negating the need for rapid pacing. Valvular function can be assessed in the fully expanded position prior to release. Partial or full recapturing/repositioning of the valve, or full retrieval, is possible at any point before uncoupling and release.

### Computed tomography assessment and implantation procedure

Computed tomography angiography (CTA) was performed on all patients before the procedure using a second generation dual-source CT (SOMATOM Definition Flash, Siemens Medical Solutions, Forchheim, Germany). All CT images were measured by a Core Imaging Lab (Corelab) based in central Europe utilizing standardized software (3mensio Medical Imaging; Pie Medical Imaging, Maastricht, The Netherlands). To evaluate the aortic valvular structures, end-systolic images were used. The final valve size for implantation was determined both by Corelab-based measurements in relation to a company-recommended sizing chart as well as operator experience. Valve oversizing in relation to the annulus was calculated as follows: (actual Lotus valve area/annular area measured by CT-1) × 100%.

All procedures were performed in a hybrid catheterization laboratory via the transfemoral approach. The procedure was performed on 20 patients (87%) with local anesthesia and on three (13%) patients with general anesthesia. A transesophageal echocardiographic (TEE) probe was placed and a temporary pacing wire was inserted into the right ventricle prior to valve implantation. The femoral access was pre-closed with two ProGlide devices (Abbott Vascular, Santa Clara, CA, USA). A preshaped TAVR 0.035-inch guidewire (Safari^2^ wire, Boston Scientific, Marlborough, MA, USA) was selected to avoid the complication of left ventricular perforation and to allow for stable advancement of the delivery system. Balloon valvuloplasty was performed according to the operator’s discretion.

The Lotus valve was implanted without rapid pacing. The valve position and PVL were assessed by TEE and aortography [20]. Three valve sizes (23 mm, 25 mm, and 27 mm) were used in this study cohort. The valve was implanted via an 18-F (23- and 25-mm device sizes) or 20-F (27-mm device) proprietary introducer sheath.

### Statistical analysis

All statistical analyses were undertaken with SPSS software (version 20; IBM Corporation, Somer, NY, USA). Continuous variables are described as mean ± standard deviation and compared with a paired Student *t *test. Categorical variables are expressed as counts and percentages and compared with a chi-square test. The first 12 Lotus TAVR procedures were categorized as early experience. A separate analysis was performed between early (*N* = 12) and late experience (*N* = 11) outcome data. A *p *value < 0.05 was considered statistically significant.

## Results

### Patient characteristics

Between November 2015 and January 2017, 23 patients underwent successful Lotus valve implantation. The mean age was 74.7 ± 5.9 years, 47.8% were female, and the mean STS score was 8.1 ± 6.6%. No patients were lost to follow-up or withdrew from the study. Nine out of 23 patients (39.1%) had BAVs (five, type 0; four, type 1). The baseline characteristics were similar between the BAV and TAV patients, except that moderate or severe aortic regurgitation (AR) was absent in the BAV group, while eight patients had significant AR in the TAV group (Table 1).

**Table 1:** Baseline characteristics of patients with TAV and BAV

	TAV (*N* = 14)	BAV (*N* = 9)	*p*
*Clinical characteristics*			
Age (years)	76.9 ± 5.7	71.2 ± 4.8	0.02
Female, *n* (%)	5 (35.7)	6 (66.7)	0.15
STS score (%)	9.8 ± 7.8	5.5 ± 3.0	0.13
NYHA class III or IV, *n* (%)	11 (78.6)	8 (88.9)	0.52
Diabetes mellitus, medically treated, *n* (%)	1 (7.14)	2 (22.2)	0.30
History of PCI or CABG, *n* (%)	0 (0)	2 (22.2)	0.07
History of atrial fibrillation, *n* (%)	3 (21.4)	1 (11.1)	0.52
History of peripheral vascular disease, *n* (%)	3 (21.4)	1(11.1)	0.52
History of cerebrovascular accident, *n* (%)	3 (21.4)	1 (11.1)	0.52
COPD, *n* (%)	1 (7.14)	3 (33.3)	0.11
Chronic renal failure, *n* (%)	3 (21.4)	2 (22.2)	0.96
*Echocardiographic assessment*			
Aortic valve area (effective orifice area; cm^2^)	0.63 ± 0.19	0.60 ± 0.15	0.67
Mean aortic valve gradient (mm Hg)	50.8 ± 8.9	52.6 ± 9.0	0.65
Peak velocity (m/s)	4.6 ± 0.6	4.7 ± 0.5	0.65
Left ventricular ejection fraction (%)	52.9 ± 11.5	54.0 ± 15.5	0.85
Aortic regurgitation (moderate or severe), *n* (%)	8 (57.1)	0 (0.0)	0.005
*Computed tomography assessment*			
Area-derived diameter (mm)	24.0 ± 1.8	24.4 ± 2.5	0.64
Perimeter-derived diameter (mm)	24.5 ± 1.8	24.9 ± 2.7	0.70
Severe aortic cusp calcification, *n* (%)	1 (7.1)	3 (33.3)	0.11

Values are mean ± standard deviation or number (%)

*BAV* bicuspid aortic valve, *CABG* coronary artery bypass graft, *COPD* chronic obstructive pulmonary disease, *NYHA* New York Heart Association, *PCI* percutaneous coronary intervention, *STS* Society of Thoracic Surgeons, *TAV* tricuspid aortic valve

### Device performance and procedural results

Vascular access and device deployment were successful for all patients. Balloon valvuloplasty was performed on 19 (82.6%) of the patients. Seven patients (30.4%) received a 23-mm Lotus valve, 12 (52.2%) received a 25-mm Lotus valve, and four received (17.4%) a 27-mm Lotus valve. During implantation in one patient who enrolled during an early phase of the study, the valve was accidentally released before confirmation of full locking, which led to unsuccessful implantation. A successful valve-in-valve procedure was immediately performed with a same-sized Lotus prosthesis. Repositioning of the Lotus valve with partial resheathing was attempted in ten patients (43.5% in all, eight in TAVs vs. two in BAVs, *p* = 0.10), and successfully redeployed in a more precise position accompanied by reduction in PVL and better hemodynamics. In one patient, a 25-mm valve was implanted initially; however, following full expansion, the prosthesis was deemed to be small with severe PVL and thus it was fully retrieved. A larger valve (27-mm Lotus) was subsequently implanted with excellent positioning and no significant PVL or complications (Table 2).

**Table 2:** Procedural characteristics and outcomes

	TAV (*N* = 14)	BAV (*N* = 9)	*p*
*Procedural characteristics (N* *=* *23)*			
General anesthesia	3 (21.4)	0 (0)	0.14
Transfemoral	14 (100)	9 (100)	–
Predilatation	13 (92.9)	6 (33.3)	0.11
Postdilatation	1 (7.1)	0 (0)	0.41
*Procedural outcomes (N* *=* *23)*			
Absence of procedural mortality	14 (100)	9 (100)	–
Successful access, delivery, deployment, delivery system retrieval	13 (92.9)	9 (100)	0.41
Successful partial valve resheathing, if attempted (*N* = 10)	8 (57.1)	2 (22.2)	0.10
Successful valve retrieval, if attempted (*N* = 1)	1 (7.1)	0	0.41
TAV-in-TAV deployment	0	1 (11.1)	0.20
Correct positioning of a single valve into proper location	13 (92.9)	9 (100)	0.41
Indexed EOA > 0.85 cm^2^/m^2a^	11 (78.6)	7 (77.8)	0.96
PGmean < 20 mm Hg or Vmax < 3m/s	11 (78.6)	8 (88.9)	0.52
No moderate/severe PVL	14 (100)	9 (100)	–

Values are number (%)

*BAV* bicuspid aortic valve, *EOA* effective orifice area, *PGmean* mean aortic valve gradient, *PVL* paravalvular leakage, *TAV* tricuspid aortic valve, *Vmax* peak velocity

^a^All were BMI < 30 kg/m^2^

### Hemodynamic and patient clinical outcomes

**Fig. 1:**
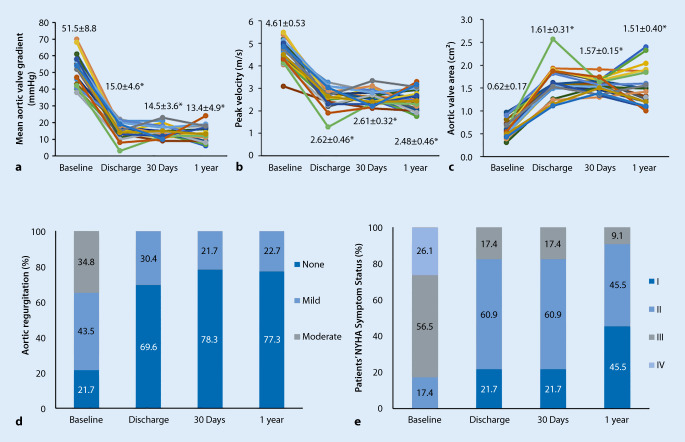
Valve hemodynamics assessed by transthoracic echocardiography and New York Heart Association (*NYHA*) functional class status. **a** Mean aortic valve gradient. **b** Peak velocity. **c** Aortic valve area. **d** Aortic regurgitation. **e** NYHA functional class status. ^*^p < 0.001 vs. baseline

Patients exhibited significantly improved valve hemodynamics from baseline to discharge, at 30 days and at 1 year (Fig. 1a–d). PGmean decreased from 51.5 ± 8.8 mm Hg at baseline to 14.5 ± 3.6 mm Hg (*p* < 0.001), Vmax decreased from 4.61 ± 0.53 m/s to 2.61 ± 0.32 m/s (*p* < 0.001), and AVA increased from 0.62 ± 0.17 cm^2^ to 1.57 ± 0.15 cm^2^ (*p* < 0.001) at 30 days. These effects were maintained at 1 year, with a PGmean value of 13.4 ± 4.9 mm Hg (*p* < 0.001 vs. baseline), a Vmax value of 2.48 ± 0.46 m/s (*p* < 0.001 vs. baseline), and an AVA value of 1.51 ± 0.40 cm^2^ (*p* < 0.001 vs. baseline). There was no difference in hemodynamic improvement between BAV and TAV patients. No moderate or severe PVL was observed, whereas mild PVL occurred in eight patients (34.8%), three of which had BAVs. The VARC-2 defined clinical outcomes at 30 days and 1 year are shown in Table 3. There were no MACCE events, life-threatening/disabling bleeding, or major vascular complications at the 30-day postimplantation follow-up, and the all-cause mortality rate at 1 year was 4.5% (one out of 23). NYHA functional class was improved by at least two levels in eight patients (34.8%) at 30 days (*p* < 0.001 vs. baseline). The improvement was maintained for at least 1 year (Fig. 1e). There was no significant difference in procedural and 30-day or 1‑year outcomes between the TAV and BAV groups (Table 4).

**Table 3:** Clinical outcomes at 30 days and 1 year

Clinical outcomes (*N* = 23)	30 days	1 year
All-cause mortality	0 (0.0)	1 (4.5)
All stroke	0 (0.0)	0 (0.0)
Urgent/emergent conversion to surgery or repeat procedure for valve-related dysfunction	0 (0.0)	0 (0.0)
Minor vascular complications^a^	1 (4.3)	1 (4.3)
Major bleeding^b^	3 (13.0)	3 (13.0)
Acute kidney injury of stage 1^c^	1 (4.3)	1 (4.3)
Periprocedural coronary obstruction (≤ 72 h)	0 (0.0)	0 (0.0)
Myocardial infarction	0 (0.0)	0 (0.0)
Conduction disturbance requiring new pacemaker	6 (26.1)	6 (26.1)

Values are number (%)

^a^There was one minor vascular complication related to access site, while no major vascular complication

^b^Not related to access, and there was no life-threatening disabling bleeding

^c^There was no acute kidney injury worse than stage 1

**Table 4:** Clinical outcomes of patients with TAV and BAV

	TAV (*N* = 14)	BAV (*N* = 9)	*p*
*Outcomes at 30 days*			
All-cause mortality, *n* (%)	0 (0.0)	0 (0.0)	–
All stroke, *n* (%)	0 (0.0)	0 (0.0)	–
Indexed EOA > 0.85 cm^2^/m^2^, *n* (%)	11 (78.6)	7 (77.8)	0.96
Mean aortic valve gradient < 20 mm Hg or peak velocity < 3 m/s, *n* (%)	11 (78.6)	8 (88.9)	0.52
No moderate/severe PVL	0 (0.0)	0 (0.0)	–
NYHA class III or IV, *n* (%)	3 (21.4)	1 (11.1)	0.52
Conduction disturbance requiring new pacemaker, *n* (%)	3 (21.4)	3 (33.3)	0.53
*Outcomes at 1 year*			
All-cause mortality, *n* (%)	1 (7.1)	0 (0.0)	0.33
All stroke, *n* (%)	0 (0.0)	0 (0.0)	–
Indexed EOA > 0.85 cm^2^/m^2^, *n* (%)	11 (78.6)	7 (77.8)	0.96
Mean aortic valve gradient < 20 mm Hg or peak velocity < 3 m/s, *n* (%)	11 (78.6)	8 (88.9)	0.52
No moderate/severe PVL	0 (0.0)	0 (0.0)	–
NYHA class III or IV, no. (%)	3 (21.4)	1 (11.1)	0.52
Conduction disturbance requiring new pacemaker, *n* (%)	3 (21.4)	3 (33.3)	0.53

Values are mean ± SD deviation or number (%)

*BAV* bicuspid aortic valve, *MACCE* major adverse cardiovascular or cerebrovascular events, *NYHA* New York Heart Association, *PVL* paravalvular leakage, *STS* Society of Thoracic Surgeons, *TAV* tricuspid aortic valve

One patient had 70% right femoral stenosis after removal of the delivery sheath; a stent was implanted accordingly without any resultant limb ischemia. Three patients (13%) had BARC(Bleeding Academic Research Consortium)-defined major bleeding, in whom no cause other than procedural-related blood loss was identified. One patient developed VARC stage 3 acute renal impairment with resolution of the creatinine to baseline on day 4.

Six patients (26.1%) required new permanent pacemaker implantation (PPI) owing to complete (third degree) atrioventricular block or complete left bundle branch block (LBBB) with symptomatic bradycardia. It is noteworthy that these six cases involved TAVs whereas no patient in the BAV group needed new PPI (50% vs. 0%, *p* = 0.006). Deeper implantation below the left coronary sinus (LCS) and greater degree of annulus oversizing were each independently associated with the occurrence of new-onset LBBB and the requirement for a new PPI. Postprocedural CT assessment revealed that the implantation depth at the LCS in patients who needed a pacemaker was significantly greater than those who did not need a pacemaker (5.75 ± 1.87 mm vs. 3.19 ± 2.24 mm, *p* = 0.02), and the difference in implantation depth also existed between TAVs and BAVs (4.88 ± 1.88 mm vs. 2.75 ± 2.47 mm, *p* = 0.03). The implantation depth in the noncoronary sinus (NCS), however, did not differ significantly between the two groups.

Three patients were declined assessment by Corelab because the annular dimensions were beyond the manufacturer recommended sizing range for the Lotus valve; two of these patients had BAVs with moderate-to-severe calcification at the annular and leaflet levels, while the other had a TAV with mild calcification continuous between the fused left and right coronary leaflets. Owing to the high surgical risk and the patients’ refusal of surgical valve replacement, it was decided to proceed with TAVR using the Lotus prosthesis following a multidisciplinary heart team assessment of CT dimensions. A 25-mm Lotus valve was implanted in one patient who had severe calcifications in the left coronary sinus; 27-mm Lotus valves were implanted in the other two patients, which equated to undersizing in relation to the annulus of −5.4%, −18.6%, and −14.1%, respectively, significantly lower than manufacturer sizing recommendation. These downsized Lotus valves were successfully deployed in the proper anatomical position with an obvious waist on the prosthesis at the annular level. Device implantation was successful in all three patients, without any complications according to the VARC-2 (Table 5).

**Table 5:** Patients declined by Corelab owing to large annulus size

Valve type	Annulus			LVOT		Lotus valve size	PPI	Echocardiographic assessments post TAVR			
	AreaD (mm)	PeriD (mm)	Calcification	AreaD (mm)	PeriD (mm)			AVA (cm^2^)	PG_mean_ (mm Hg)	Peak velocity (m/s)	PVL
Bicuspid (type 1)	27.8	28.8	Moderate to severe	29.5	30.8	27	no	1.55	11	2.3	no
Bicuspid (type 0)	27.7	28.1	Moderate to severe^a^	29.5	30.3	25	no	1.74	10	2.2	no
Tricuspid (functional bicuspid)	29.1	29.4	Moderate	29.3	29.6	27	no	1.53	15	2.7	no

*AR* aortic regurgitation, *AreaD* area-derived diameter, *AVA* aortic valve area, *LVOT* left ventricular outlet tract, *PeriD* perimeter-derived diameter, *PGmean* mean aortic valve gradient, *PPI* permanent pacemaker implantation, *PVL* paravalvular leakage, *TAVR* transcatheter aortic valve replacement

^a^Severe calcification especially in left coronary sinus

## Discussion

This is the first Chinese study to evaluate the acute and 1‑year safety, efficacy, and clinical outcomes of the Lotus TAVR system in symptomatic high-risk surgical patients with severe aortic valve stenosis, while also comparing BAV and TAV patient subgroups. There were no deaths or strokes within 30 days, no residual moderate or severe PVL, and no significant differences in clinical outcomes between BAV and TAV patients.

The reported prevalence of BAV deformities is 0.5%–2% [21]. Patients presenting for TAVR in the Chinese population have a very high frequency of BAV morphology, close to 50%, which far exceeds the frequency in the Western world, and with an enormous calcium burden, which presents challenges for TAVR in this population [22]. TAVR for bicuspid AS was regarded as a relative contraindication in early guidelines owing to the unfavorable anatomical characteristics, such as annular eccentricity, asymmetric leaflet calcifications, and dilated ascending aorta, which led to a higher risk of paravalvular regurgitation, annulus rupture, aortic dissection, or other poorer outcomes after the procedure [23–29]. The Lotus valve is designed to minimize PVL with its unique Adaptive Seal technology. In addition, the ability to recapture and reposition allows the operator to fully assess valve performance and the degree of PVL in its fully expanded form before release. The clinical outcomes of TAVR with the Lotus valve in patients with bicuspid AS are suggested to be superior to those following implantation of early-generation devices [16, 30, 31]. In the current study, the nine patients with bicuspid AS showed similar procedural outcomes to the 14 patients with tricuspid AS. This finding confirms that the Lotus valve is safe and efficient for the treatment of bicuspid AS.

Trials utilizing the Lotus aortic valve replacement system have consistently shown an incidence of new PPI that is higher when compared with balloon-expandable valves, although similar to that following implantation of self-expanding valves [32–39]. The reported new PPI rate of 26.1% in this study is comparable to that reported previously (REPRISE I: 36%; REPRISE II: 28.6%; RESPOND: 30.0%; RELEVANT: 30.7%; [40–43]). The pacing rate reduced significantly as operator experience increased. After realizing that the conduction disturbance that may result in new PPI was associated with valve implantation depth and the degree of annulus oversizing during the learning curve, we modified our valve sizing protocol and minimized the implantation depth for late-experience patients. The lower incidence of new-onset LBBB and new PPI partly confirmed the relevance of these findings. A large-scale study is warranted for further confirmation.

According to the recommendations in the Boston Scientific Lotus sizing charts, patients with an annulus size of > 27 mm are not considered to meet the minimal anatomical requirements as per the patient evaluation criteria, and would be declined a Lotus valve. In our experience, this is not necessarily the case. By undersizing of the valve, this group of patients may still benefit from TAVR using the Lotus device, suggesting that additional evaluation criteria beyond annular diameters and calcification scores need to be combined in the decision-making.

### Study limitations

The fact that a small number of patients were enrolled, a randomized control group was absent, and the follow-up duration was short are limitations of this study. Moreover, there was no independent Corelab to analyze the echocardiographic data and to control the quality of the clinical data collection and accuracy.

## Conclusion

Our single-center experience showed that the Lotus valve is feasible and effective for Chinese patients with aortic valve stenosis and atypical anatomy, including BAVs and a large aortic annulus. However, a large-scale study is still warranted in the Chinese population to fully evaluate the efficacy and safety of the Lotus valve system.
